# Supplementary material for: Development of a computer-aided design software for the quantitative evaluation of aesthetic damage
Source: PLoS One. 2019 Dec 18;14(12):e0226322. doi: 10.1371/journal.pone.0226322 (PMC6919621; doi:10.1371/journal.pone.0226322)
Supplement: S1 Code site — 1. (ZIP) [file pone.0226322.s001.zip › [EESC_jr][Projeto]Relatorio_07_21/reports/1/2017.07.25/09:06:01/report.pdf]

Preâmbulo

Na data de 25/07/2017 no horário 10:00, o(a) Perito(a) Sr. Rafael Yonezawa de Mello, Estudante, designado(a)para proceder ao exame pericial solicitado em: Rafael de Mello, do sexo Masculino, nascido(a) em 10/07/1997, ocupação Estudante, portador(a) do RG 44.173.380-3 SSP-SP, cujo endereço Avenida Henriqueta Soares, passa a expor a avaliação de dano estético corporal.

Exame objetivo da lesão

A lesão ora objeto de análise teve como natureza Acidente de trabalho ocorrido em 05/06/2017. O prejuízo estético consiste em lesão cicatricial consolidada na região: Rosto, Costas no lado superior, direito.

Trata-se de cicatriz Quelóide de tamanho/frequência Única: Retilínea de orientação Horizontal de pigmentação Normocrômica medindo 1 centímetro(s) na horizontal, 5 centímetros na vertical e 5 milímetros de Profundidade. De contornos Regulares. A lesão é visível a 3 (três) metros nas relações sociais. A lesão tem repercussão na vida diária afetiva conforme fotos anteriores e atuais..

Conclui-se que classificada segundo os parâmetros de dano estético AIPE - Análise do Impacto do Prejuízo Estético (escala de seis níveis e pontuação entre 0 e 50 pontos), como sendo dano estético relativo à área 1, a lesão é caracterizada por 19 pontos e grau de prejuízo estético: Importante.

Escore final: 19;
Grau de prejuízo: Importante.

São Carlos, SP, 25/07/2017

____

Sr. Rafael Yonezawa de Mello
